# Genomic Analysis of Heterosis in an Angus × Hereford Cattle Population

**DOI:** 10.3390/ani13020191

**Published:** 2023-01-04

**Authors:** El Hamidi Hay, Andy Roberts

**Affiliations:** Fort Keogh Livestock and Range Research Laboratory, Agricultural Research Service, USDA, Miles City, MT 59301, USA

**Keywords:** crossbreeding, beef cattle, heterosis, GWAS, dominance

## Abstract

**Simple Summary:**

Crossbreeding is commonly practiced in the US. Traditionally, it has been used to improve performance by mating animals from different breeds to benefit from heterosis and complementarity (i.e., the general tendency for crossbred offspring to have superior performance compared to those of the parents). With the advent of genomic information, we are now able to dissect heterosis on a molecular level. In this study, we evaluated the effects of heterozygosity on growth traits in an Angus × Hereford cross-population and identified regions in the genome associated with heterosis. The effects of crossbreeding were an increase of 0.03 kg, 5.13 kg, 6.02 kg in birth weight, weaning weight and yearling weight, respectively. Moreover, regions in the genome having significant effects related to heterosis were associated with birth weight, weaning weight and yearling weight and were located on chromosomes 1, 2, 6, 21, 14, 19, 13 and 12. Genes in these regions were reported to be involved in growth and other important physiological mechanisms. Our study revealed several regions associated with dominance effects and contributing to heterosis. These results could be beneficial in optimizing crossbreeding.

**Abstract:**

Crossbreeding is widely used in the beef cattle industry to exploit its several benefits. This study evaluated the effects of heterozygosity on growth traits in an Angus × Hereford cross-population. Moreover, a genome wide association study was conducted to detect regions in the genome with significant dominance effects on growth traits contributing to heterosis. A total of 1530 animals comprising of pure Line 1 Hereford, Angus and Angus × Line 1 Hereford cross. Growth phenotypes included birth weight, weaning weight and yearling weight. All animals were genotyped with GeneSeek GGP LD 50k. Significant effects of genomic heterozygosity on growth traits were detected. These effects were 0.03 kg (0.006), 5.13 kg (0.04), 6.02 kg (0.08) on birth weight, weaning weight and yearling weight, respectively. Genome wide association study revealed several SNP markers with significant heterotic effects associated with birth weight, weaning weight and yearling weight. These SNP markers were located on chromosomes 1, 2, 6, 21, 14, 19, 13 and 12. Genes in these regions were reported to be involved in growth and other important physiological mechanisms. Our study revealed several regions associated with dominance effects and contributing to heterosis. These results could be beneficial in optimizing crossbreeding.

## 1. Introduction

Crossbreeding is commonly practiced in the beef cattle industry. Crossbreeding allows producers to exploit heterosis and combine beneficial characteristics from different breeds for desirable performance [1,2]. To improve performance in certain environments crossbreeding has been used to match adapted animals with other breeds with desirable traits [3,4]. Several studies have shown that crossbreeding results in an amelioration of various traits such as milk yield, weaning weight and average daily gain [5,6,7]. Heterosis or hybrid vigor is the difference of the crossbred progeny over the average of its purebred parents [1,3]. The first generation cross (F1) benefits from 100% of heterosis and decays in successive generations [5,8]. A recent study by JA Basarab, J Crowley, MK Abo-Ismail, G Manafiazar, EC Akanno, VS Baron and G Plastow [9] used genomic information to evaluate heterosis in a crossbred beef cattle population. The results showed an increase of 51 ± 20 d longer survival and an increase of 35.7 ± 15.2 kg in calf weaning weight with a 10% heterosis which resulted in an overall $161 gain per heifer in a year.

Heterosis is controlled by non-additive effects such as dominance, over dominance and epistatic genetic effects [10,11]. However, in most genetic evaluation programs, additivity of genes is assumed, and the non-additive effects are ignored [12]. Few studies have shown the potential benefits of including non-additive effects, specifically dominance effects [12,13,14]. Using genomic information, S Bolormaa, JE Pryce, Y Zhang, A Reverter, W Barendse, BJ Hayes and ME Goddard [13] quantified the percentage of variance explained by dominance for economically important traits in beef cattle and found it to range from 0 to 42%. In a recent study, EC Akanno, MK Abo-Ismail, L Chen, JJ Crowley, Z Wang, C Li, JA Basarab, MD MacNeil and GS Plastow [14], dominance was found to explain up to 9% of the total variation in beef cattle growth and carcass traits. 

With the advancement in high-throughput technology, genomic information such as single nucleotide polymorphism (SNP) is readily available. Genome wide association studies (GWAS) allow the usage of SNP to detect genomic regions associated with phenotypes of interest [15]. Furthermore, GWAS provide an opportunity to understand mechanisms such as heterosis and dominance at the molecular level and optimize crossbreeding. 

The beef cattle populations used in this study consisted of Line 1 Hereford, Angus and crosses of both. The Line 1 Hereford cattle is a unique population, founded over 80 years ago and maintained as a closed herd at USDA-ARS, Fort Keogh Livestock and Range Research Laboratory, Miles City, MT, USA [16,17,18]. Management specific to this herd has been previously described in several studies [17,19,20]. Historically, the selection objective in Line 1 was focused on increasing post weaning gain [21]. Since 2011, the selection was primarily focused on improving calving ease (direct and maternal) while maintaining weaning and yearling weights to at least herd averages. In year 2013, an Angus herd was founded and crossed with Line 1 Hereford which generated F1 animals and advanced crosses with varying percentages.

The objectives of this study are to first quantify the effects of heterozygosity on growth traits using SNP information and second to detect genomic regions exhibiting dominance effects associated with heterotic effects. 

## 2. Materials and Methods

All research protocols were approved by a local Animal Care and Use Committee.

### 2.1. Data: Animals, Genotypes, and Phenotypes

The dataset consisted of 722 Line 1 Hereford animals, 608 Angus and approximately 350 Line 1 × Angus crossbred animals with varying percentages. The growth phenotypes considered were birth weight (BW), weaning weight (WW) and yearling weight (YW). 

Animals were genotyped with varying SNP density panels. Quality control of genotype data consisted of filtering out SNPs with a call rate less than 90%, minor allele frequency (MAF) less than 5%, a heterozygous deviation greater than 15% from Hardy–Weinberg Equilibrium. Animals with a call rate lower than 90% were also eliminated. Animals genotyped with low density chips were imputed to the low density of 50K marker panel using FImpute software [22]. Missing genotypes for animals with low density SNP panel were imputed with FImpute software using population and pedigree information (Sargolzaei et al., 2011). The average allelic *R*2 was 0.94 which indicates high imputation accuracy of the missing genotypes. After quality control and imputation, the total number of SNP genotypes were 28,320.

### 2.2. Statistical Analysis

**Figure 1:**
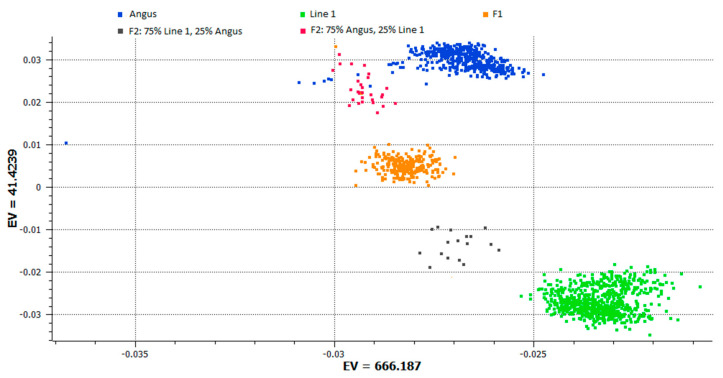
Principal component analysis plot showing the distribution of animals using the first two principal components of the genomic relationship matrix.

A principal component analysis was conducted using genomic information in order to investigate the population structure as shown in (Figure 1). 

Genomic heterozygosity was calculated as the total number of heterozygous loci divided by the total number of SNP and the following model was implemented to assess the effect of heterozygosity on growth traits:

(1) $$y_{ijk}=\mu +sex_{ij}+CG_{ik}+\beta_{1}H_{i}+e_{ijk}\;\;$$

where $y_{ijk}$ is the observation for animal *i* belonging to sex class *j* and contemporary group CG class *k* (grouped by year and age-of-dam subclasses), $\beta_{1}$ is the regression coefficient on the individual level of genomic heterozygosity $H_{i}$ and $e_{ijk}$ is the residual assumed to be independent and normally distributed. For weaning and yearling weight, observations were adjusted to a standardized age. 

### 2.3. Genome-Wide Association Analyses

EMMAX was implemented using SNP & Variation Suite 7 (Golden Helix, Inc., Bozeman, MT, USA), this is a single-locus mixed model GWAS approach that fits a genomic relationship matrix to account for genetic covariance among animals. An additive and a dominance model were implemented separately. The false discovery rate (FDR) was set to 0.05 to correct for multiple testing in SNP & Variation Suite 7 (Golden Helix, Inc., Bozeman, MT, USA). The following model was used:

(2) $$y=\mu +\overset{n}{\underset{j=1}{\sum }}z_{j}\alpha_{j}+e\;$$

where ***y*** is the vector of corrected phenotypes, ***µ*** is the overall mean, ***n*** is the number of SNPs, *z**_j_*** is the genotype covariate of the *j*th SNP coded according to the additive model (0, 1, and 2) α*_j_* is the allelic substitution effect of SNP_j_, and e is the vector of random residuals. To test dominance a second model was implemented similar to the above model except SNP markers coding was modified to 1 for the heterozygous genotype AB and 0 for the two homozygous genotypes AA and BB for each SNP and α*_j_* is the dominance effect. 

## 3. Results and Discussion

Plotting the first two principal components showed five distinct groups. The plot clearly shows the divergence between the breeds and the crosses with varying breed percentages (Figure 1). The groups are Line 1 Hereford, Angus, 50% Angus 50% Line 1 Hereford, 25% Angus 75% Line 1 Hereford, and 75% Angus 25% Line 1 Hereford. 

**Figure 2:**
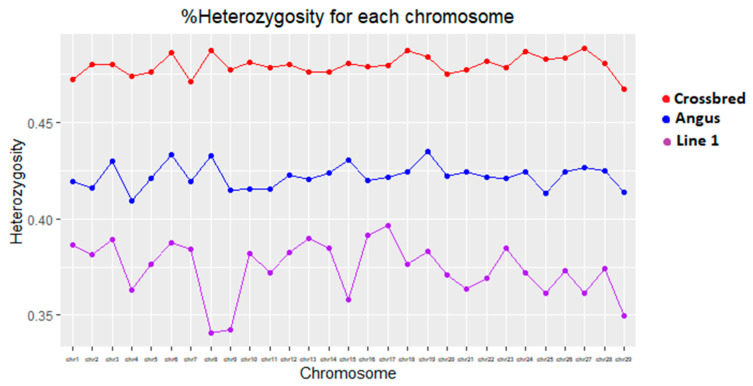
Percentage of heterozygosity per chromosome using SNP information for all breeds used in the study.

To evaluate the effects of crossbreeding on growth traits, the amount of heterozygosity was calculated using genomic information and regressed on the phenotypes. Figure 2 shows the amount of heterozygosity. As expected, the crossbreds had the highest amount of heterozygosity (47.1%), followed by Angus (41.8%) and Line 1 Hereford (36.9%) had the least. A study by WS Pitchford, JM Pitchford, JG Alexopoulos and ML Hebart [23] reported a heterozygosity of 41.1% for Angus calves and 44.6% for Hereford calves. The amount of heterozygosity in Angus was similar to the estimated heterozygosity for the Angus animals in this study. On the other hand, Hereford heterozygosity is higher than the line 1 Hereford estimated in this study. This is sensible since the Line 1 Hereford has been maintained as a closed population for many years. 

Studies by GM Smith, D Laster and KE Gregory [24], KE Gregory, LV Cundiff, GM Smith, D Laster and H Fitzhugh Jr [25], KE Gregory, GM Smith, L Cundiff, R Koch and D Laster [26] and LV Cundiff, K Gregory and R Koch [27] showed that calves from Hereford cows were heavier at birth than calves from Angus cows. This is in concordance with birthweights observed in this study despite the high inbreeding in the Hereford Line 1 (Table 1). Furthermore, crossbred animals had the highest birthweights which could be explained by retained heterosis. A composite breed developed by the USDA/ARS Meat and Animal Research Center referred to as MARCIII resulted in higher birth weights compared to Angus and Hereford [28]. For weaning and yearling weights, Angus calves had higher weights than Hereford Line 1 and the crossbreds which is similar to the findings reported by E Casas, R Thallman and L Cundiff [29].

The regression of phenotypes on heterozygosity showed an increase in birth weight by 0.03 ± 0.006 kg (*p* < 0.0001) for each percentage unit increase in heterozygosity. Similarly, weaning, and yearling weights increased by 5.13 ± 0.04 kg (*p* < 0.0001), 6.02 ± 0.08 kg (*p* < 0.0001), respectively (Table 2). Previous studies have reported positive heterosis effects for weaning weight and post-weaning gain in crosses of British and Continental cattle breeds [30,31]. A recent study by EC Akanno, L Chen, MK Abo-Ismail, J Crowley, Z Wang, C Li, J Basarab, M MacNeil and G Plastow [32] using genomic information to estimate heterosis in Angus, Charolais, and Hereford crosses showed a significant increase in (*p* < 0.05) for average daily gain and yearling weight. On the other hand, no significant effect was found for birth weight and weaning weight. The differences in heterozygosity effects on growth seen in this study compared to EC Akanno, L Chen, MK Abo-Ismail, J Crowley, Z Wang, C Li, J Basarab, M MacNeil and G Plastow [32], could be due to the genetic variation within breeds; Line 1 Hereford is highly inbred and crossing it with Angus resulted in high heterozygosity. 

### 3.1. Genome-Wide Association

The genome wide association study of additive and dominance effects of growth traits yielded several significant regions (Figure 3, Figure 4 and Figures S1–S6). Interestingly, the dominance model yielded more significant SNP than the additive model. 

### 3.2. Additive Effects

This study yielded several SNP markers with additive effects significantly associated with growth traits (Table 3), some of which were previously reported and some novel. For example, a significant region on chromosome 20 in 4.5 Mb associated with birth weight was previously reported to be associated with growth and birth weight in several beef cattle breeds [33]. This region contains genes ATP6V0E1, ERGIC1, RPL26L1 which are involved in metabolic and cellular processes. The other significantly associated regions were novel. In region 46 MB, chromosome 26, genes CLRN3, FOXI2, MIR2285C, PTPRE are located which are involved in developmental and metabolic processes. 

For weaning weight, all significant regions were novel, however the same regions were found to be associated with reproduction and carcass traits. A significant region on chromosome 1 in 100 MB was reported to be associated with carcass traits specifically meat color [34]. A GWAS study in Holstein revealed this same region to be also associated with milk production and reproduction [35]. Furthermore, this region harbors genes involved in mitochondrial activity and anaerobic metabolism. An additional significant region associated with weaning weight was detected on chromosome 4 in 41 Mb. This region was also reported to be associated with carcass traits [36]. 

For yearling weight, a region on chromosome 20 in 14 Mb was significant and was previously reported to be associated with body weight and growth [37]. An additional significant region on chromosome 1 in 138 Mb was reported to be associated with postnatal growth traits [38]. 

### 3.3. Dominance Effects

For dominance effects, several regions were found to be significantly associated with the three growth traits (Table 4). For birth weight, a significant region on chromosome 2 in 112 Mb associated with birth weight was previously reported to be associated with postnatal growth and birth weight [33]. This region harbors genes: AP1S3, CUL3, DOCK10, FAM124B, MRPL44, SERPINE2, WDFY1 which are involved in various metabolic processes. For weaning weight, a significant on region on chromosome 4 in 16 Mb associated with this trait was previously reported to be associated with fatness in Angus [36].Further, a region on chromosome 6 in 15 Mb and a region on chromosome 21 in 3.5 Mb were significantly associated with yearling weight. These regions were previously reported to be associated with body weight and growth in Simmental, Red Angus and Shorthorn breeds [37]. Furthermore, these same regions were reported in a recent GWAS of dominance effects by EC Akanno, L Chen, MK Abo-Ismail, JJ Crowley, Z Wang, C Li, JA Basarab, MD MacNeil and GS Plastow [39] to have a significant heterotic effect on birth weight, weaning weight and daily gain. Surveying these regions on chromosome 6 and 21 showed to harbor several genes (CHSY1, GABRA5, GABRB3, bta-mir-2888, CASP6, CFI, ELOVL6, ENPEP, GAR1, LRIT3, MIR2444, PITX2, PLA2G12A, RRH) involved in glucose and lipid metabolism and body fat mass. 

## 4. Conclusions

In this study, we identified several SNPs with significant dominance associations with growth traits in a crossbred beef cattle population. The identified potential candidate genes within significant SNP markers are involved in growth-related physiological mechanisms. However, these SNPs warrant further investigation to determine their use in optimizing crossbreeding programs and their ability to predict heterosis.

## Figures and Tables

**Figure 3:**
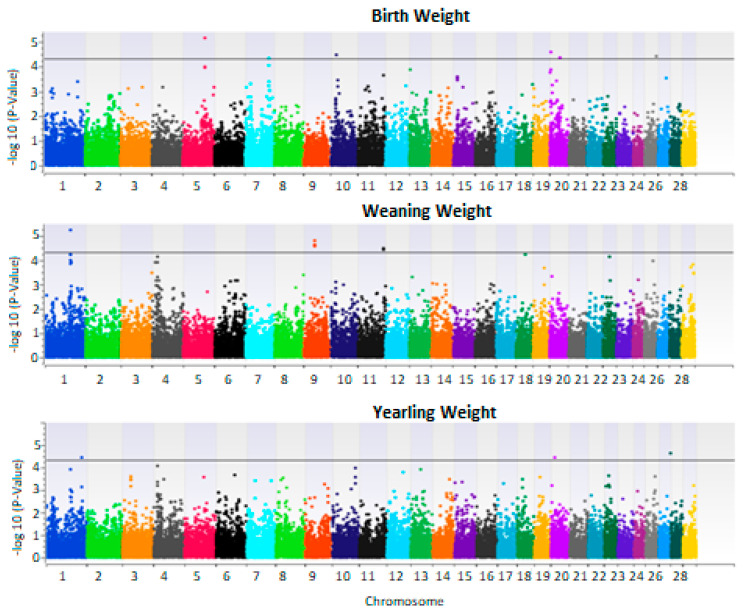
Manhattan plot of genome wide association analysis results of additive effects for birth using EMMAX, weaning and yearling weights. X-axis represents chromosomal locations and Y-axis is −log10 of *p*-values. The black line is −log10 (5e−5).

**Figure 4:**
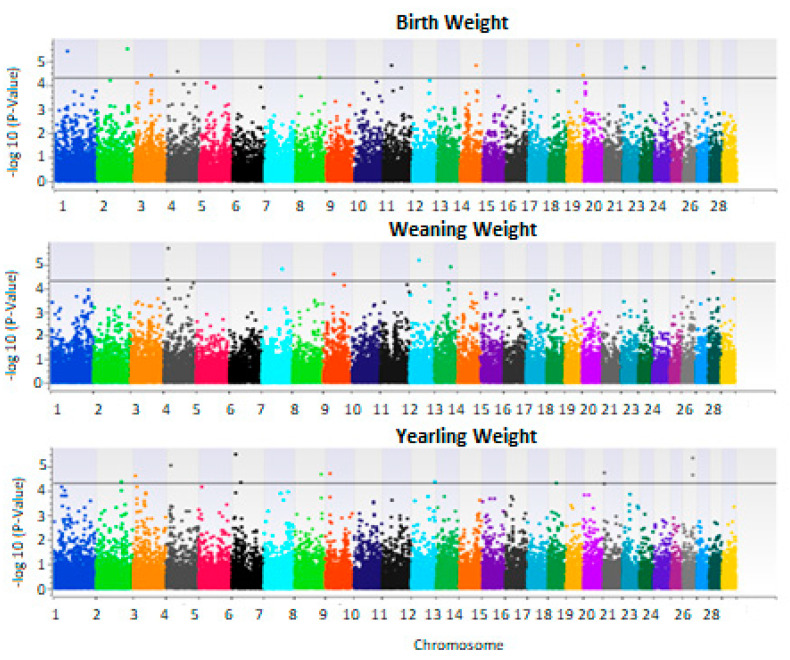
Manhattan plot of genome wide association analysis results of dominance effects using EMMAX for birth, weaning and yearling weights. X-axis represents chromosomal locations and Y-axis is −log10 of *p*-values. The black line is −log10 (5e−5).

**Table 1:** Summary statistics of growth phenotypes.

Breed	Trait	n	Mean (SD)
Line 1	Birth Weight (kg)	722	36.92 (4.75)
Line 1	Weaning Weight (kg)	718	194.41 (37.29)
Line 1	Yearling Weight (kg)	718	298.42 (93.19)
Angus	Birth Weight (kg)	608	35.20 (5.29)
Angus	Weaning Weight (kg)	608	231.91 (42.24)
Angus	Yearling Weight (kg)	589	347.96 (56.2)
Angus * × Line1	Birth Weight (kg)	215	37.82 (2.31)
Angus * × Line 1	Weaning Weight (kg)	199	209.19 (24.10)
Angus * × Line 1	Yearling Weight (kg)	169	335.69 (16.65)
Angus * × Line 1 F2	Birth Weight (kg)	121	35.58 (2.62)
Angus * × Line 1 F2	Weaning Weight (kg)	119	224.56 (18.37)
Angus * × Line 1 F2	Yearling Weight (kg)	90	306.67 (11.28)
Line 1 * × Angus	Birth Weight (kg)	345	38.26 (2.41)
Line 1 * × Angus	Weaning Weight (kg)	335	225.46 (23.98)
Line 1 * × Angus	Yearling Weight (kg)	220	332.33 (26.96)
Line 1 * × Angus F2	Birth Weight (kg)	84	37.08 (1.26)
Line 1 * × Angus F2	Weaning Weight (kg)	119	229.41 (11.75)
Line 1 * × Angus F2	Yearling Weight (kg)	90	311.68 (5.23)

* Sire breed.

**Table 2:** Linear effects of genomic heterozygosity on growth traits.

Trait	H * Effect	*p*-Value
Birth Weight (kg)	0.03 (0.006)	0.0001
Weaning Weight (kg)	5.13 (0.04)	0.0001
Yearling weight (kg)	6.02 (0.08)	0.0001

* Heterozygosity calculated from SNP data.

**Table 3:** Results of significant additive SNP effects from genome wide association study of growth traits.

Trait	SNP	Chromosome	Position	−log10 (*p*-Value)
BW	BovineHD0500023948	5	84635051	5.15
	Hapmap57038-rs29015626	20	4567765	4.58
	BovineHD1000006398	10	19462017	4.45
	BovineHD2600013437	26	46999583	4.40
	BovineHD2000010948	20	38376008	4.35
	BovineHD0700027153	7	92917772	4.33
WW	BovineHD0100028626	1	100258442	5.23
	BovineHD0900011585	9	41673609	4.79
	ARS-BFGL-NGS-114241	11	100472048	4.48
YW	BovineHD2800000001	28	5302	4.62
	Hapmap20495-rs29024564	1	138692649	4.43
	BovineHD2000004465	20	14291458	4.41

**Table 4:** Results of significant dominance SNP effects from genome wide association study of growth traits.

Trait	SNP	Chromosome	Position	−log10 (*p*-Value)
BW	BovineHD1900011884	19	41700252	5.67
	BovineHD0200032395	2	112608207	5.51
	BovineHD0100013823	1	49017600	5.41
	BovineHD1100009030	11	30187133	4.82
	BovineHD1400016579	14	59818285	4.81
	BTA-99960-no-rs	22	8765709	4.74
	ARS-BFGL-NGS-32774	23	13371915	4.71
	Hapmap60225-rs29010786	4	37385962	4.56
	UA-IFASA-7751	3	62689947	4.42
	BovineHD1900017738	19	61764496	4.40
	BovineHD0800026507	8	89344276	4.33
WW	BovineHD0400004973	4	16939402	5.68
	BovineHD1200009887	12	33674092	5.17
	ARS-BFGL-NGS-7266	13	59514859	4.90
	BovineHD0700022399	7	76343644	4.80
	BovineHD2800004761	28	16946164	4.63
	BovineHD0900011239	9	40542770	4.57
	Hapmap57378-rs29020063	29	42749808	4.57
YW	ARS-BFGL-NGS-119888	6	15252879	5.49
	BTB-00942520	26	37956801	5.33
	BovineHD0400004973	4	16939402	5.02
	BovineHD2100000540	21	3518772	4.73
	Hapmap44304-BTA-87076	9	18372617	4.71
	BovineHD0800029668	8	100388737	4.65
	BovineHD0300002944	3	8918173	4.62
	BovineHD1200024816	12	85647505	4.36
	Hapmap53672-rs29025245	2	94314937	4.36
	BovineHD0600009773	6	55115455	4.33

## Data Availability

The data supporting the findings of this study are available upon request from the author El Hamidi Hay: elhamidi.hay@usda.gov and with permission from the USDA Agricultural Research Service.

## Supplementary Materials

The following are available online at https://www.mdpi.com/article/10.3390/ani13020191/s1, Figure S1. Q-Q plot of genome wide association analysis results of additive effects for birth weight. Figure S2. Q-Q plot of genome wide association analysis results of additive effects for weaning weight. Figure S3. Q-Q plot of genome wide association analysis results of additive effects for yearling weight. Figure S4. Q-Q plot of genome wide association analysis results of dominance effects for birth weight. Figure S5. Q-Q plot of genome wide association analysis results of dominance effects for weaning weight. Figure S6. Q-Q plot of genome wide association analysis results of dominance effects for yearling weight.
