# Supplementary material for: Genomic Analysis of Heterosis in an Angus × Hereford Cattle Population
Source: Animals (Basel). 2023 Jan 4;13(2):191. doi: 10.3390/ani13020191 (PMC9854582; doi:10.3390/ani13020191)
Supplement: Supplementary file 1 [file animals-13-00191-s001.zip › animals-1969148-supplementary.pdf]

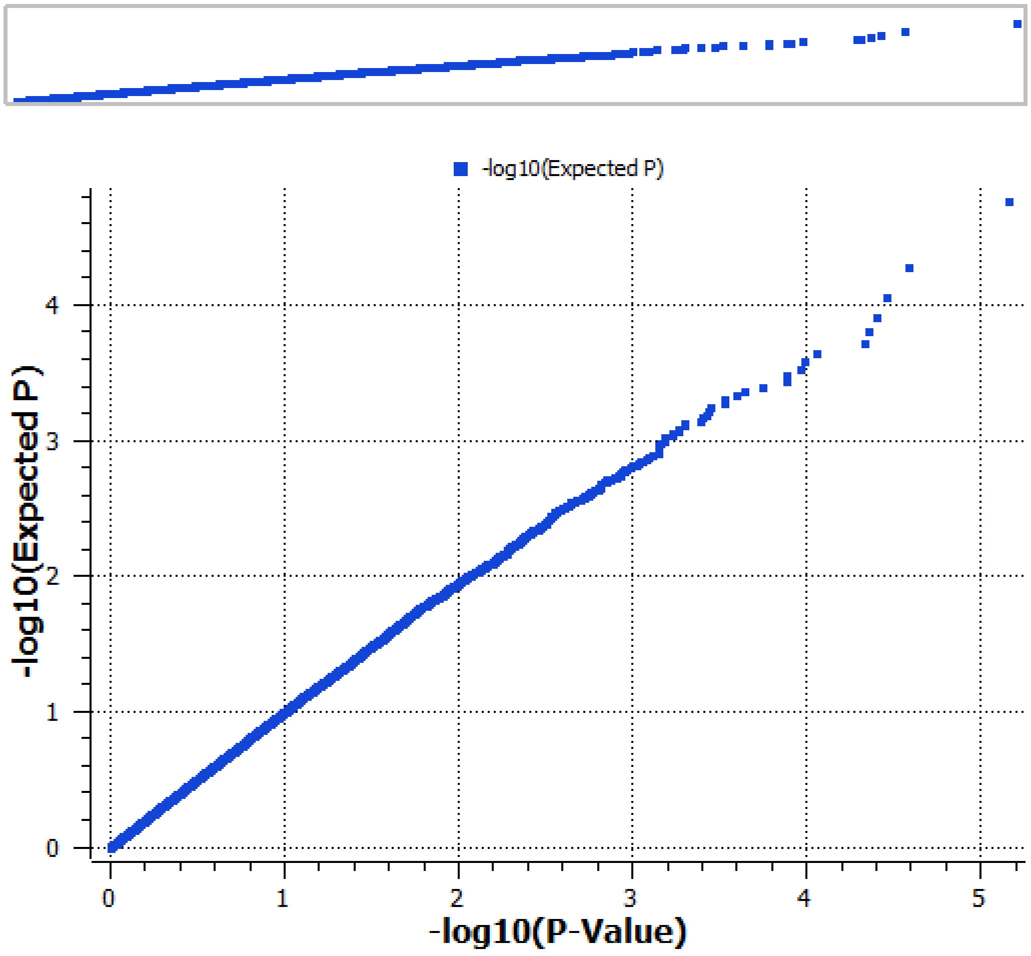

Figure S1. Q-Q plot of genome wide association analysis results of additive effects for birth weight

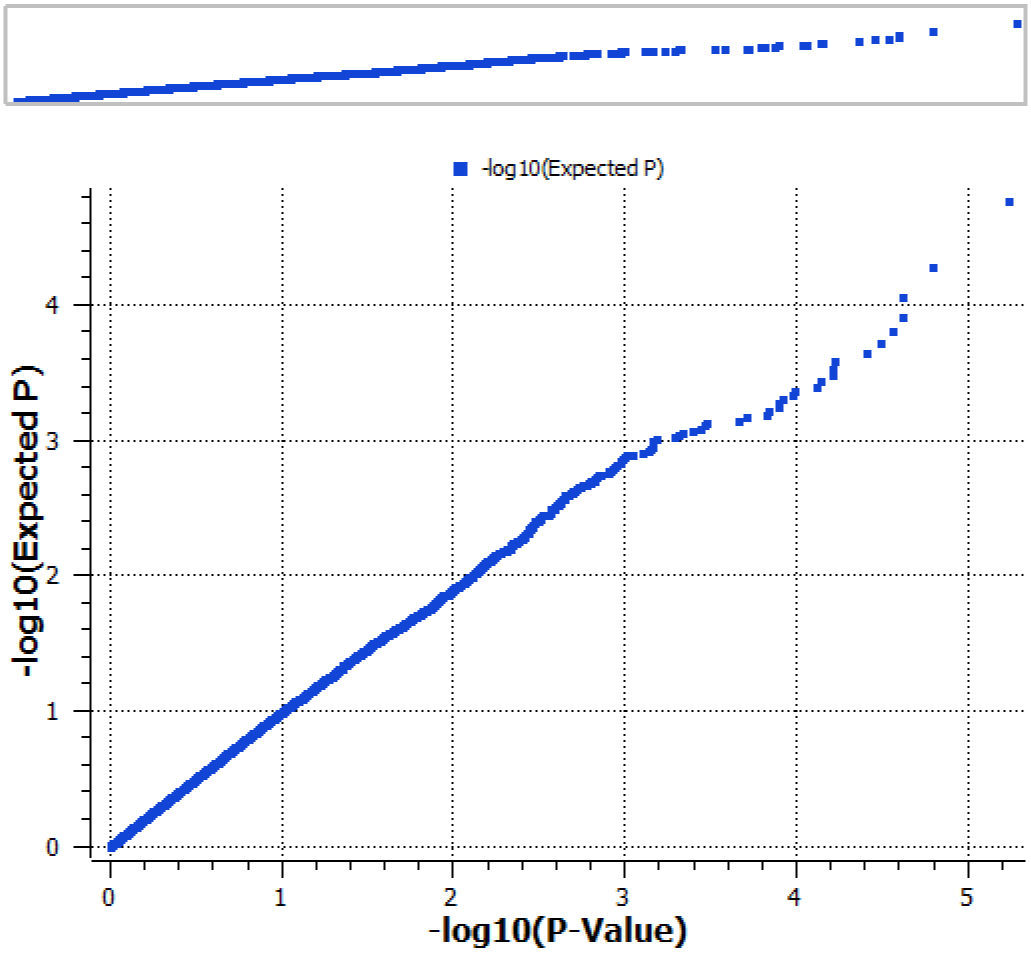

Figure S2. Q-Q plot of genome wide association analysis results of additive effects for weaning weight

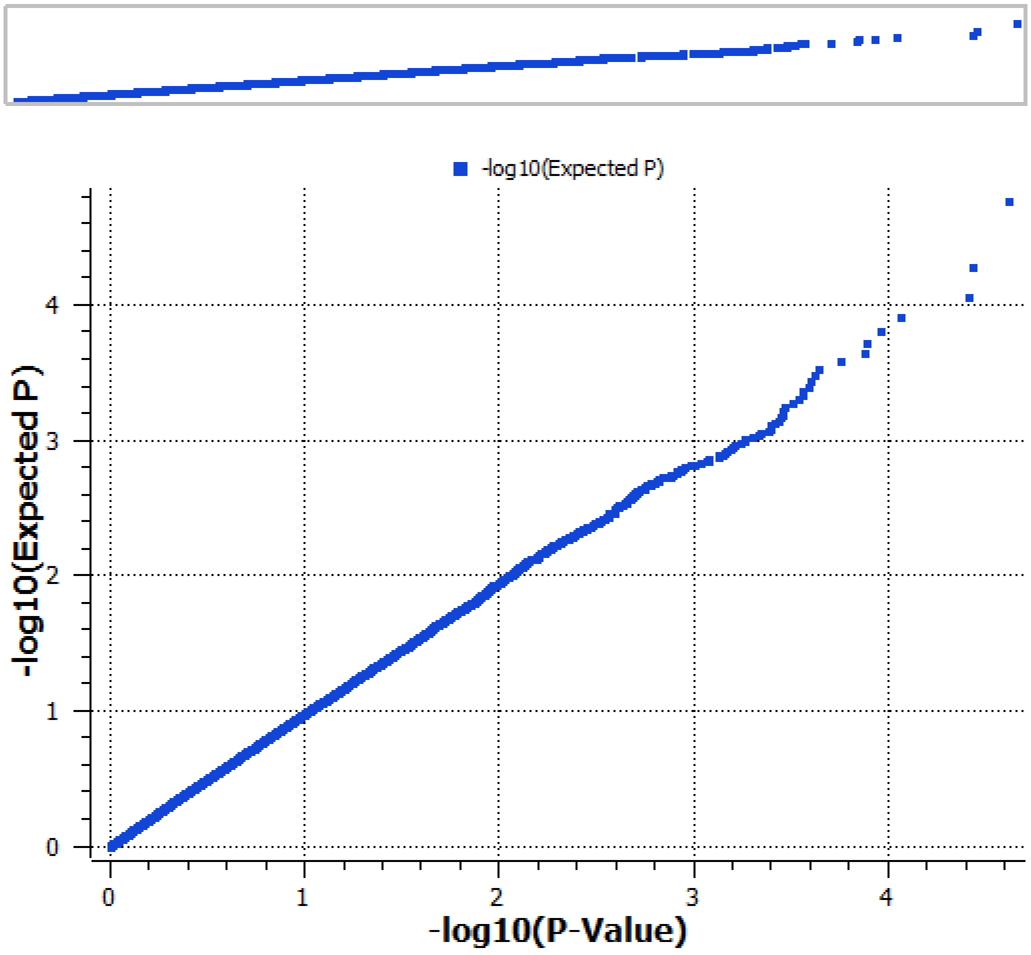

Figure S3. Q-Q plot of genome wide association analysis results of additive effects for yearling weight

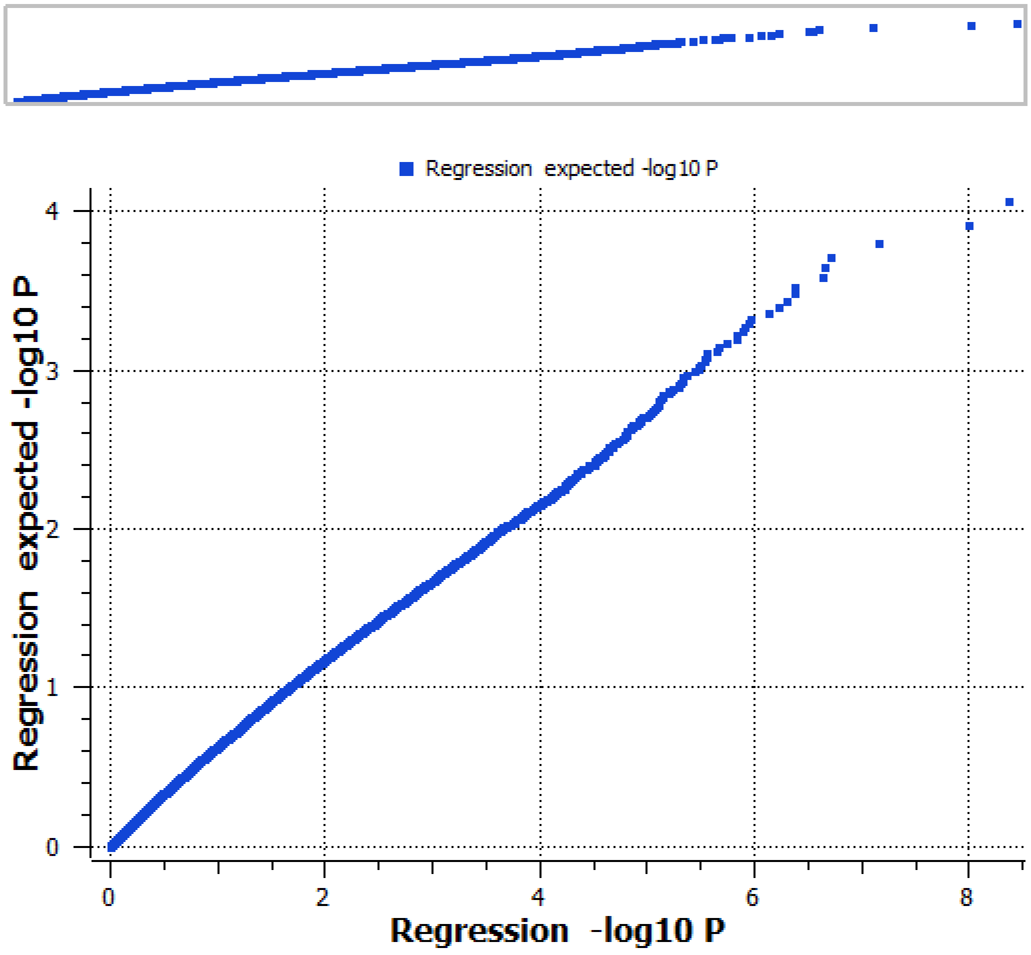

Figure S4. Q-Q plot of genome wide association analysis results of dominance effects for birth weight

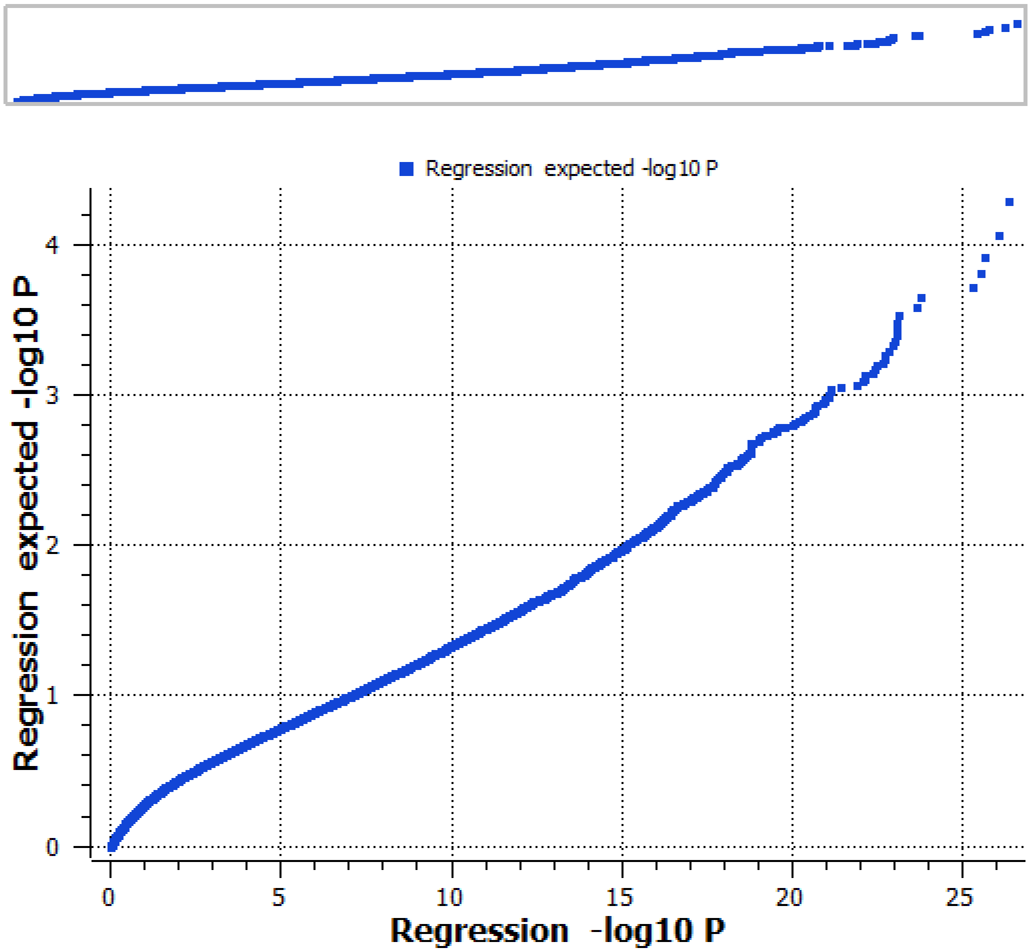

Figure S5. Q-Q plot of genome wide association analysis results of dominance effects for weaning weight

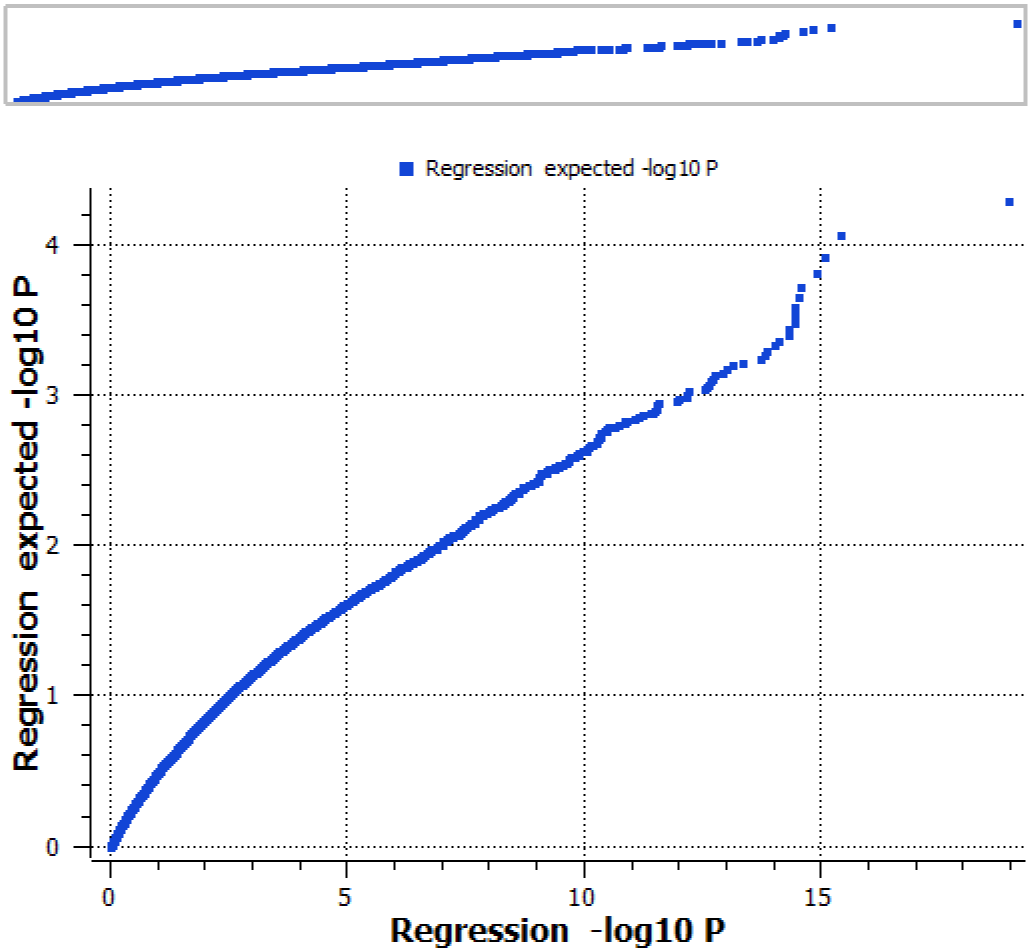

Figure S6. Q-Q plot of genome wide association analysis results of dominance effects for yearling weight
